# Inhalation of Nitrous Oxide

**Published:** 1847-08

**Authors:** 


12. *Inhalation of Nitrous Oxide.*—E. E. Marcy, M.D., in the Boston Med. and Surg. Journal, reports the removal of a large schirrous testicle, without pain, under the influence of the nitrous oxide. The following extract shows his estimate of the agent.

The legislature of Connecticut has passed a vote of thanks to Dr. Horace Wells, of Hartford, for his great discovery, which consists in the use of "*Nitrous Oxide Gas or Ether in Surgical Operations.*"

"Another superiority which it possesses over the ether is, that its after effects are far less unpleasant—less headache, less lassitude, and less depression of the nervous system, always resulting from its use. Ether generally causes troublesome choking and cough; the gas scarcely ever. Ether is objectionable on account of the unpleasant smell which it communicates to the room; the gas possesses no disagreeable odor. Ether abstracts largely from the oxygen of the arterial blood, thus becoming a direct source of disease; the gas has no such effect. Ether gives rise to pains in the head, lassitude, impaired vital energy, and other symptoms indicating a serious depression of the nervous system; the gas rarely produces any of these effects, and if ever, only in a slight degree. In order to produce the full effect of the ether it is customary to reduce the patient to a state of stupor; the gas is capable of rendering the body entirely insensible to the pain of the most severe surgical operation, without putting the patient to sleep, or causing any stupor! We have often observed patients watch the progress of severe operations upon their own persons, with countenances as smiling and happy as if they were enjoying a delightful treat."

